# Metformin Reduces Oxidative Damage in RNASEH2-Mutant Aicardi-Goutières Cells

**DOI:** 10.3390/genes16080922

**Published:** 2025-07-30

**Authors:** Francesca Dragoni, Jessica Garau, Bartolo Rizzo, Simona Orcesi, Costanza Varesio, Rosalinda Di Gerlando, Matteo Bordoni, Eveljn Scarian, Cristina Cereda, Orietta Pansarasa, Stella Gagliardi

**Affiliations:** 1 IRCCS Mondino Foundation, 27100 Pavia, Italy; francesca.dragoni@mondino.it (F.D.); jessica.garau@mondino.it (J.G.); b.rizzo@golgicenci.it (B.R.); simona.orcesi@mondino.it (S.O.); costanza.varesio@gmail.com (C.V.); rosalinda.digerlando@mondino.it (R.D.G.); matteo.bordoni@mondino.it (M.B.); eveljn.scarian@mondino.it (E.S.); stella.gagliardi@mondino.it (S.G.); 2 Department of Brain and Behavioral Sciences, University of Pavia, 27100 Pavia, Italy; 3 Department of Biology and Biotechnology “L. Spallanzani”, University of Pavia, 27100 Pavia, Italy; 4 Department of Pediatric, Buzzi Children’s Hospital, 20154 Milan, Italy; cristina.cereda@asst-fbf-sacco.it; 5 Department of Biomedical and Clinical Sciences, University of Milan, 20122 Milan, Italy

**Keywords:** metformin, Aicardi-Goutières Syndrome, RNASEH2, oxidative stress, ROS, mitochondria

## Abstract

Background: Aicardi-Goutières Syndrome (AGS) is a rare neuroinflammatory condition characterized by early-onset symptoms that extend outside the nervous system. Due to the rarity of the disease, the pathogenesis is not well understood, and its diagnosis and treatment remain elusive. We recently demonstrated mitochondrial abnormalities and increased reactive oxygen species (ROS) levels in lymphoblastoid cell lines (LCLs) derived from *RNASEH2B*- and *RNASEH2A*-mutated AGS patients. On this background, we turned our attention to metformin, the first-choice drug for type 2 diabetes, as a possible treatment acting on oxidative stress in RNASEH2-mutant AGS cells. Methods and Results: By means of flow cytometry, we found that metformin treatment significantly decreases ROS production in *RNASEH2B*- and *RNASEH2A*-mutated AGS LCLs. Of note, metformin treatment reduces the green JC-1 monomeric signal and, concurrently, increases the red JC-1 signal in both mutated LCLs, accounting for restoration of the mitochondrial membrane potential. Immunofluorescence staining shows a decrease in 8-oxoG levels only in *RNASEH2B*- mutated AGS LCLs. Finally, the significant upregulation of Forkhead Box O3 (*FOXO3*), cytochrome C somatic (*CYCS*), and superoxide dismutase 2 (*SOD2*) mRNA levels in *RNASEH2B*-mutated AGS LCLs after metformin treatment points to *FOXO3* signaling as a possible mechanism to reduce oxidative stress. Conclusions: In conclusion, even if these pilot results need to be confirmed on a larger cohort, we shed light on metformin treatment as a valid approach to ameliorate oxidative stress-related inflammation in AGS patients.

## 1. Introduction

Aicardi-Goutières Syndrome (AGS) is a severe, early-onset neuroinflammatory condition characterized by a rare genetic basis and a broad spectrum of symptoms extending beyond the central nervous system. Its global prevalence is estimated to be approximately 1 in 100,000 live births, though this might be an underestimate due to diagnostic challenges and clinical heterogeneity [1,2]. Onset typically occurs in infancy or early childhood, often presenting within the first year of life, though milder, later-onset forms have also been described [3]. The prognosis for AGS is highly variable, ranging from severe neurological impairment leading to early death to milder forms with significant developmental delays but longer survival [2,3]. It is defined by the erroneous production of a type I interferon-mediated immune response. The condition is severe and has a high related morbidity and fatality rate in the majority of instances [3,4].

The pathological phenotype encompasses diverse neurological manifestations, including cortical blindness, seizures, spasticity, dystonia, microcephaly, and psychomotor delay. Moreover, a long-standing association exists between AGS and other systemic pathological traits such as chilblains-like skin lesions, glaucoma, hypothyroidism, elevated autoantibody levels [4], and lupus-like disease [5].

AGS exhibits primarily an autosomal recessive inheritance pattern, although dominant forms, often associated with specific gene mutations (e.g., *IFIH1*, *ADAR1*), have also been identified [6]. To date, mutations in nine genes (*TREX1*, *RNASEH2B*, *RNASEH2C*, *RNASEH2A*, *ADAR1*, *SAMHD1*, *IFIH1*, *LSM11*, and *RNU7*-1) have been identified in AGS patients and linked to its pathophysiology. These genes are localized across various chromosomes: *TREX1* on 3p21.31, *RNASEH2B* on 13q14.3, *RNASEH2C* on 11q13.4, *RNASEH2A* on 13q14.3, *ADAR1* on 1q21.3, *SAMHD1* on 20q11.23, *IFIH1* on 2q24.3, *LSM11* on 1p31.1, and *RNU7*-1 on 12q13.13 [2,3,6,7]. *RNASEH2A*, *RNASEH2B*, and *RNASEH2C* encode the three subunits of RNase H2 protein, and they are the most commonly mutated genes in AGS patients. In eukaryotes, this enzyme is the primary source of cellular ribonuclease activity, and it may play a role in numerous phases of nucleic acid metabolism [2,6,7]. All of these genes are critically involved in the metabolism and sensing of nucleic acids (NAs) [8,9,10,11]. A hallmark of AGS is the persistently elevated levels of interferon-alpha (IFN-α) in both serum and cerebrospinal fluid [6], alongside increased expression of IFN-α-stimulated genes (ISGs) in peripheral blood, collectively termed the “interferon signature” [8,10,11].

Current therapeutic approaches for AGS are largely symptomatic and supportive, focusing on managing neurological and systemic manifestations to improve patient comfort and quality of life. These include physical and speech therapy to address motor and communication difficulties and nutritional support [5]. Management of specific complications such as glaucoma, scoliosis, and thyroid dysfunction is also critical [12].

More recently, strategies targeting the underlying neuroinflammatory pathway, particularly the aberrantly activated type I interferon response, have shown promise. Janus kinase (JAK) inhibitors, such as baricitinib, have emerged as a significant development, demonstrating the ability to reduce inflammation and, in some cases, lead to clinical improvements and developmental gains in AGS patients [5,13,14]. Other immunomodulatory therapies, including glucocorticoids, intravenous immunoglobulin (IVIG), and antiretroviral therapies (RTIs), have been explored empirically, aiming to reduce the detrimental effects of chronic inflammation [15]. However, the efficacy of these interventions can be variable, highlighting the urgent need for more targeted and genotype-specific treatment strategies.

Given these compelling evidences, AGS is now definitively classified as an inflammatory disease, making the role of reactive oxygen species (ROS) in its pathomechanism particularly pivotal. Elevated ROS levels typically lead to an imbalance in cellular redox homeostasis, culminating in substantial oxidative stress predominantly driven by mitochondrial impairment [16]. In this context, AGS appears to be no exception. Our previous work in 2022 described significant mitochondrial abnormalities in lymphoblastoid cell lines (LCLs) derived from AGS patients carrying mutations in the *RNASEH2B* and *RNASEH2A* genes [17]. We specifically demonstrated a significant increase in ROS production and oxidative stress-induced DNA damage in *RNASEH2B*-mutated patients. Furthermore, we confirmed the increased release of mitochondrial DNA (mtDNA) in *RNASEH2B*-mutated LCLs as a potential inducer of oxidative stress and the inflammatory state [18].

Among the most promising pharmacological agents capable of inhibiting ROS production is metformin. Metformin (1,1-dimethylbiguanide hydrochloride), the first-line oral drug for type 2 diabetes, has recently gained attention as a potential therapeutic agent for various conditions, including cancers, aging, cardiovascular dysfunction, neurodegenerative disorders, and polycystic ovary syndrome [19]. Recent studies have demonstrated that metformin can induce the activation of *FOXO3*, thereby reducing ROS/reactive nitrogen species (RNS) levels in immune cells [19]. Moreover, metformin treatment has been shown to mediate pharmacological actions in an AMP-activated protein kinase (AMPK)-independent manner, including induction of neurotrophic factors, reduction of neuroinflammation, and prevention of abnormal protein aggregation, all contributing to its neuroprotective effects [20,21]. Additional research highlights metformin’s multifaceted actions toward the mitochondrial respiratory chain, AMPK, the AMP:ATP ratio, cyclins, and cellular homeostatic mechanisms such as autophagy, apoptosis, and redox balance [22]. Metformin also exhibits the capacity to restore mitochondrial activity, reversing organelle fragmentation and remodeling cristae abnormalities [20].

Considering our previous findings [17], the present study investigates the beneficial impact of metformin treatment in reducing ROS overproduction and mitigating the associated oxidative stress condition in LCLs derived from AGS patients carrying mutations in the *RNASEH2B* and *RNASEH2A* genes, as well as from healthy controls.

## 2. Materials and Methods

### 2.1. Patient Enrolment

Patients diagnosed with AGS were enrolled at the IRCCS Mondino Foundation in Pavia, Italy. A healthy volunteer (female, age 23) with no history of pharmaceutical therapy or pathology was recruited from the Immunohematological and Transfusional Service at the IRCCS Policlinico S. Matteo Foundation in Pavia, Italy.

Prior to sample collection and testing, informed consent was obtained from the parents of all subjects involved in the study and the healthy control. The study was approved by the local ethics committee (approval n. 3549/2009 of 30 September 2009 and 11 December 2009, and n. 20170035275 of 23 October 2017), IRCCS Mondino Foundation, Pavia, Italy.

The cohort included a 13-year-old female patient with a compound heterozygous mutation in the *RNASEH2A* gene (p.R108W + p.F231L), indicating that one mutation was inherited from each parent, and a 9-year-old male patient with a homozygous mutation in the *RNASEH2B* gene (p.A177T). Table 1 provides a summary of the main clinical and demographic data for these AGS patients. Diagnoses were determined based on established clinical criteria.

Blood samples from AGS patients were taken in EDTA-containing vacutainers. A semi-automated Maxwell^®^ 16 DNA Purification System (Promega, Madison, WI, USA) was used to extract genomic DNA. Both a Qubit^®^ fluorometer and a NanoDrop ND1000 UV-Vis Spectrophotometer (Thermo Scientific, Waltham, MA, USA) were used to quantify the DNA. An Illumina MiSeq Sequencer was used for DNA processing and sequencing analysis. MiSeq software (Real Time Analysis RTA v.1.18.54 and Casava v.1.8.2, Illumina, Inc., San Diego, CA, USA) was used to load the samples onto the MiSeq instrument and carry out the first steps of bioinformatic analysis, such as base calling and demultiplexing. Annovar software was used to perform variant annotation (table_annovar.pl). According to an in silico software program (SIFT, PolyPhen, MutationTaster), mutations were deemed pathogenic if they were infrequently found in healthy control databases (such as the dbSNP and 1000 Genomes databases), predicted to change the encoded protein sequence (such as nonsynonymous, nonsense, splice-site, frameshift, and insertion/deletion mutations), and expected to negatively impact protein function. Sanger sequencing was used to confirm all identified variants and genetic regions with low coverage (less than 30×) [7].

### 2.2. Cell Isolation

Peripheral blood mononuclear cells (PBMCs) were isolated from 9 mL of peripheral venous blood using Histopaque^®^-1077 (Sigma-Aldrich, St. Louis, MO, USA) according to the manufacturer’s instructions. Briefly, blood samples were layered onto an equal volume of Histopaque^®^-1077 in a centrifuge tube and spun for 30 min at 300× *g* with low deceleration.

Following centrifugation, PBMCs were carefully collected from the intermediate phase. They were then washed in 1X PBS (Sigma-Aldrich, St. Louis, MO, USA), and the supernatant was discarded after a 10 min centrifugation at 200× *g*. For cryopreservation, cells were resuspended in freezing medium (FBS supplemented with DMSO) and stored in liquid nitrogen.

### 2.3. EBV Immortalization and Cell Culture

Given the rarity of this pathological condition and the inherent difficulties in obtaining fresh blood samples from pediatric patients in a manner compatible with experimental timelines, we opted to immortalize our cells. B lymphocytes were isolated from PBMCs from the AGS patients and healthy control, then immortalized via Epstein-Barr virus (EBV) infection. EBV immortalization was performed by Dr. Chiara Baldo at the Laboratorio di Genetica Umana, IRCCS Istituto Giannina Gaslini, Genoa, Italy. For this research, we utilized LCLs derived from an *RNASEH2A* (p.R108W + p.F230L) and an *RNASEH2B* (p.A177T)-mutated patient, as well as a healthy control. These cell lines were cultured in RPMI 1640 medium (Carlo Erba Reagents S.r.l., Cornaredo, Italy) supplemented with 20% fetal bovine serum (FBS) (Sigma-Aldrich, St. Louis, MO, USA), 0.3 mg/L L-glutamine (Sigma-Aldrich, St. Louis, MO, USA), and 5% penicillin-streptomycin (Sigma-Aldrich, St. Louis, MO, USA). The cells were maintained at 37 °C in a humidified environment with 5% CO_2_.

For routine maintenance, the cells were pelleted by centrifugation, washed in 1X PBS, and then provided with fresh culture medium. When not in active use, the cells were resuspended in freezing medium (FBS supplemented with DMSO) for cryopreservation in liquid nitrogen.

### 2.4. Metformin Treatment

Metformin hydrochloride (S1950, Selleckchem, Houston, TX, USA) was dissolved in H_2_O to create a 120 mM stock solution. This stock was then added to the cell growth medium to achieve a final working concentration of 500 µM. To ensure consistent drug exposure, fresh metformin was added every 24 h for a total treatment period of 72 h, as reported in the literature [20]. We confirmed that cell viability remained consistent throughout the treatment period. For the non-treated (NT) control cells, an equivalent volume of fresh medium was added at the same intervals.

### 2.5. ROS Production Flow Cytometry Analysis

ROS production was quantified using a DCFDA/H2DCFDA-Cellular ROS Assay Kit (ab113851, Abcam, Cambridge, UK) following the manufacturer’s recommendations. Briefly, 1 × 10^5^ live cells were harvested and stained with the cell-permeant dye 2′,7′-dichlorofluorescein (DCFDA) for 30 min at 37 °C. After incubation, the cells were centrifuged for 8 min at 1600× *g*, and the resulting pellet was resuspended in 300 µL of 1X PBS. The cell suspensions were then analyzed using a BD FACS Canto II flow cytometer, and the raw data were processed with BD FACSDiva software v.9.0 (BD Biosciences, Franklin Lakes, NJ, USA).

### 2.6. JC-1 Mitochondrial Membrane Potential Assay

Mitochondrial membrane potential (MMP) in LCLs from healthy subjects and AGS-mutated patients was directly measured using the fluorescent dye 5,5,6,6′-tetrachloro-1,1′,3,3′-tetraethylbenzimidazoylcarbocyanine iodide (JC-1), as provided in the Mitochondrial Membrane Potential Assay Kit (ab113850, Abcam, Cambridge, UK), following the manufacturer’s instructions. In healthy cells possessing fully functional mitochondria, the lipophilic cationic dye JC-1 readily enters the active, negatively charged mitochondrial matrix in a concentration-dependent manner. There, it forms JC-1 aggregates, which emit a distinct red fluorescence (appearing as discrete spots). Conversely, in cells where the mitochondrial membrane potential has been compromised, only a limited amount of the JC-1 dye can accumulate within the mitochondria. As a consequence, the JC-1 dye remains in its monomeric form, resulting in diffusive green fluorescence.

Briefly, approximately 2 × 10^5^ cells were centrifuged and washed in 500 µL of 1X Dilution Buffer. Following centrifugation, cells were resuspended in the JC-1 working solution and incubated at 37 °C. After an additional washing step, the cells were plated for fluorescence analysis using an Axio Imager 2 fluorescence microscope (Zeiss, Germany) equipped with an Axiocam Mrm camera (Zeiss, Jena, Germany). Images capturing both red (emission at 590 ± 17.5 nm) and green (emission at 530 ± 15 nm) fluorescence intensities were acquired.

The corrected total cell fluorescence (CTCF) was quantified using ImageJ software v. 1.54p and calculated according to established protocol using the formula CTCF = Integrated density − (Area of selected cell × Mean fluorescence of background readings).

### 2.7. Immunofluorescence (IF)

Approximately 1 × 10^5^ live cells were seeded onto slides (Thermo Fisher Scientific, Waltham, MA, USA) and incubated at RT for 30 min. The cells were then fixed with a 4% paraformaldehyde solution at RT, followed by permeabilization with 0.1% Triton™ X-100 (Sigma-Aldrich, St. Louis, MO, USA). The samples were treated with a blocking solution (0.05% Triton™ X-100, 1% BSA in 1X PBS) before incubation with primary antibodies for 2 h at RT. Subsequently, the cells were incubated with secondary antibodies for 1 h at RT. Finally, the cells were washed with 1X PBS, mounted with ProLong^®^ Gold Antifade Reagent with DAPI (Thermo Fisher Scientific, Waltham, MA, USA), and air-dried, and a coverslip was mounted and sealed with nail polish. The following primary antibodies were used for immunofluorescence: Rabbit monoclonal anti-TOM20 antibody (#42406, Cell Signaling Technology, Danvers, MA, USA, dilution 1:250); Mouse monoclonal anti-8oxoG antibody (sc-130914, Santa Cruz Biotechnology, Dallas, TX, USA, dilution 1:250). Secondary antibodies included CFTM 594 goat anti-mouse (Sigma-Aldrich, St. Louis, MO, USA, dilution 1:1000) and CFTM 488A goat anti-rabbit (Sigma-Aldrich, St. Louis, MO, USA, dilution 1:1000).

CTCF was quantified using ImageJ software and calculated according to established protocols using the formula CTCF = Integrated density − (Area of selected cell × Mean fluorescence of background readings).

### 2.8. RNA Extraction with Trizol Reagent

RNA was extracted from both treated and NT LCLs using Trizol^®^ reagent (Sigma-Aldrich, St. Louis, MO, USA) following the manufacturer’s instructions. Subsequently, RNA quantification was performed using a NanoDrop ND1000 UV-Vis Spectrophotometer (Thermo Fisher Scientific, Waltham, MA, USA).

### 2.9. Reverse Transcription

For reverse transcription, 1000 ng of RNA was used per sample. This was performed with the iScript™ Reverse Transcription Supermix kit for RT-qPCR (Bio-Rad, Richmond, CA, USA) following the manufacturer’s recommendations.

### 2.10. Real-Time PCR

For analysis of *FOXO3*, *CYCS*, and *SOD2* expression, qPCR reactions were prepared containing 200 nM of each oligonucleotide, 7.5 μL of SYBR Green SuperMix (Bio-Rad, Richmond, CA, USA), and 1 μL of cDNA template or H_2_O control. Primers were designed using the Primer3Plus tool (https://www.primer3plus.com/index.html, accessed on 1 September 2024) and aligned using Primer-BLAST (https://www.ncbi.nlm.nih.gov/tools/primer-blast/index.cgi?GROUP_TARGET=on, accessed on 1 September 2024). Their sequences are detailed in Table 2. Cycle threshold (Ct) values were automatically recorded for each replicate qPCR reaction. Mean Ct values were then normalized against those determined for the β-actin gene, which served as the housekeeping gene. Fold-expression differences relative to healthy controls were calculated using the 2^−ΔΔCt^ method. qPCR was performed using a CFX96™ Real-Time PCR Detection System (Bio-Rad, Richmond, CA, USA) with the following thermal cycling protocol: 1 cycle at 95 °C for 5 min, followed by 40 cycles of 95 °C for 15 s and 60 °C for 45 s.

### 2.11. Statistical Analysis

Statistical analyses were performed and figures were generated using GraphPad PRISM version 9 (San Diego, CA, USA). Each analysis was conducted with biological triplicates. Data normality was assessed using the Shapiro–Wilk test, and all datasets passed this test. For all analyses, a two-tailed Student’s *t*-test was employed, with data expressed as means ± SEM. A *p*-value of less than 0.05 was considered statistically significant. Below are listed all the statistical comparisons made.

For ROS assessment, the following statistically significant differences were observed:

- *RNASEH2B*-mutated metformin-treated (Met) vs. *RNASEH2B*-mutated NT: Difference between means ± SEM = −32.45 ± 5.794, *p* = 0.005
- *RNASEH2B*-mutated NT vs. healthy control NT: Difference between means ± SEM = 14.98 ± 4.895, *p* = 0.0376

Statistical analysis for JC-1 fluorescence, reflecting mitochondrial membrane potential, yielded the following significant results:

JC-1 green fluorescence:

- *RNASEH2B*-mutated Met vs. *RNASEH2B* mutated NT: Difference between means ± SEM = −1.202 ± 0.1408, *p* < 0.0001
- *RNASEH2A*-mutated Met vs. *RNASEH2A*-mutated NT: Difference between means ± SEM = −0.1415 ± 0.05777, *p* = 0.0184
- *RNASEH2B*-mutated NT vs. healthy control NT: Difference between means ± SEM = 1.356 ± 0.1448, *p* < 0.0001
- *RNASEH2A*-mutated NT vs. *RNASEH2B*-mutated NT: Difference between means ± SEM = −1.313 ± 0.1528, *p* < 0.0001

JC-1 red fluorescence:

- *RNASEH2B*-mutated Met vs. *RNASEH2B*-mutated NT: Difference between means ± SEM = 2.028 ± 0.3729, *p* < 0.0001
- *RNASEH2A*-mutated Met vs. *RNASEH2A*-mutated NT: Difference between means ± SEM = 2.291 ± 0.3635, *p* < 0.0001
- *RNASEH2B*-mutated NT vs. healthy control NT: Difference between means ± SEM = −1.349 ± 0.3187, *p* = 0.0001
- *RNASEH2A*-mutated NT vs. healthy control NT: Difference between means ± SEM = −1.301 ± 0.3309, *p* = 0.0003

For 8-oxoG assessment, indicative of DNA oxidative stress damage, the following statistically significant differences were noted:

- *RNASEH2B*-mutated Met vs. *RNASEH2B*-mutated NT: Difference between means ± SEM = −0.4827 ± 0.06887, *p* < 0.0001
- *RNASEH2B*-mutated NT vs. healthy control NT: Difference between means ± SEM = 0.3145 ± 0.08019, *p* = 0.0003
- *RNASEH2A*-mutated NT vs. *RNASEH2B*-mutated NT: Difference between means ± SEM = −0.3225 ± 0.06903, *p* < 0.0001

Statistical analysis for gene expression by real-time PCR revealed the following significant findings:

- *SOD2*, *RNASEH2B*-mutated Met vs. *RNASEH2B*-mutated NT: Difference between means ± SEM = 1.998 ± 0.6572, *p* = 0.0228
- *CYCS*, *RNASEH2B*-mutated Met vs. *RNASEH2B*-mutated NT: Difference between means ± SEM = 0.4212 ± 0.1402, *p* = 0.0239
- *FOXO3*, *RNASEH2B*-mutated Met vs. *RNASEH2B*-mutated NT: Difference between means ± SEM = 0.4853 ± 0.1473, *p* = 0.0165

## 3. Results

### 3.1. Metformin Treatment Reduces ROS Generation

We started by using flow cytometry to measure ROS production in LCLs derived from patients with *RNASEH2B* and *RNASEH2A* mutations associated with AGS.

**Figure 1:**
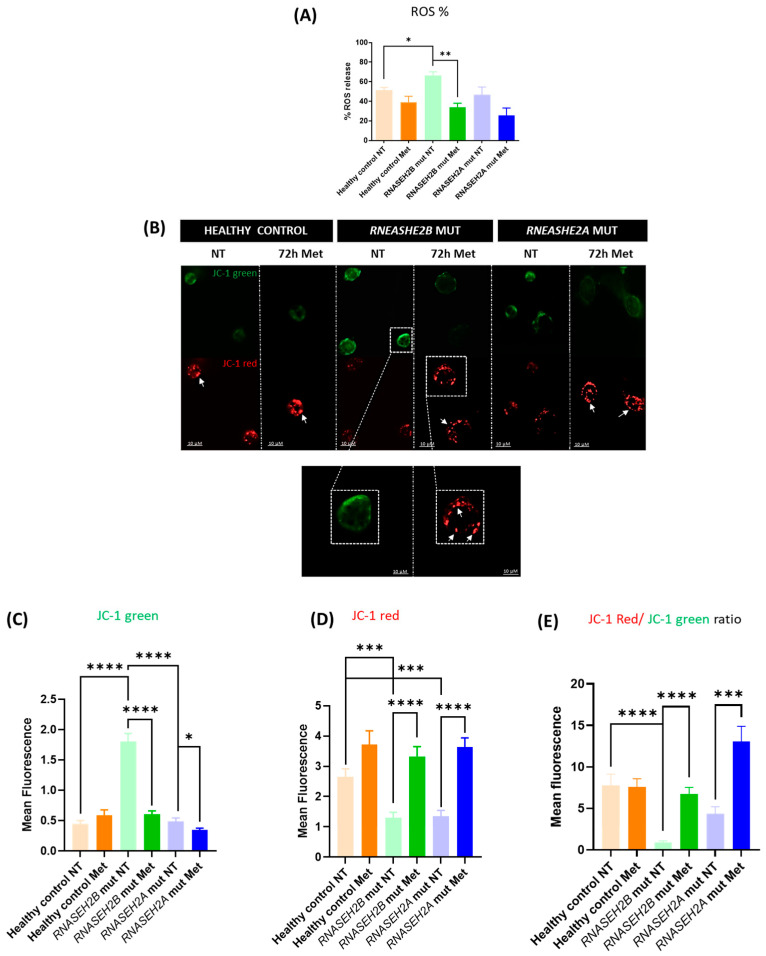
Mitochondrial ROS production and membrane potential of RNASEH2-mutated LCLs and healthy control LCLs. (**A**) Flow cytometry analysis of ROS production in AGS patients with mutations in the *RNASEH2B* and *RNASEH2A* genes and a healthy control with (72 h Met) or without (NT) 72 h metformin treatment. The bar graph represents the mean of three biological experiments. Two-tailed Student’s *t*-test was performed, and data are expressed as means ± SEM; * *p* < 0.05; ** *p* < 0.01. (**B**–**D**) Mitochondrial membrane potential assay and mean fluorescence of LCLs derived from *RNASEH2B*- and *RNASEH2A*-mutated AGS patients and a healthy control, with (72 h Met) or without (NT) 72 h metformin treatment. Green JC-1 monomers represent depolarized mitochondrial membrane potential. Red JC-1 aggregates represent normal mitochondrial membrane potential. White arrows indicate JC-1 spots. (**E**) We reported the JC-1 red fluorescence/JC-1 green fluoresce ratio. The bar graph represents the mean of three biological experiments. Three cells per image were quantified, and three images per condition were analyzed. Two-tailed Student’s *t*-test was performed, and data are expressed as means ± SEM; * *p* < 0.05; *** *p* < 0.001; **** *p* < 0.0001.

As depicted in Figure 1A, and consistent with our prior research [17], the percentage of ROS production was significantly higher (*p* = 0.0376) in *RNASEH2B*-mutated NT LCLs compared to both healthy control NT LCLs and *RNASEH2A*-mutated NT LCLs. Notably, *RNASEH2A*-mutated NT LCLs did not exhibit any significant difference in ROS generation when compared to healthy control NT LCLs.

Intriguingly, following 72 h of metformin treatment, we observed a decreasing trend in ROS generation across all cell lines. This reduction reached statistical significance (*p* = 0.005) in *RNASEH2B*-mutated Met LCLs when compared to their NT counterparts (Figure 1A). These findings strongly suggest a beneficial effect of metformin treatment in ameliorating the oxidative stress observed in LCLs from AGS patients.

### 3.2. Metformin Treatment Restores Mitochondrial Membrane Potential in RNASEH2B- and RNASEH2A-Mutated LCLs from AGS Patients

In our previous work, published in 2022, we demonstrated that LCL lines derived from AGS patients exhibit alterations in MMP [17]. Building upon this finding, we utilized IF to investigate whether 72 h metformin treatment could restore physiological MMP in LCLs from AGS-mutated patients (Figure 1B–D).

As showed in Figure 1B, the green JC-1 signal was highly diffuse in *RNASEH2B*-mutated NT LCLs. This diffusive pattern was significantly greater compared to both healthy control NT LCLs (*p* < 0.0001) and *RNASEH2A*-mutated NT LCLs (*p* < 0.0001), unequivocally indicating an altered MMP (Figure 1B,C). In contrast, while the green JC-1 signal in *RNASEH2A*-mutated NT LCLs appeared diffuse, its detected intensity was similar to that observed in healthy control NT LCLs.

Crucially, after 72 h of metformin treatment, the JC-1 monomeric green signal was reduced in both treated mutant LCLs. This reduction was statistically significant in *RNASEH2A*-mutated Met LCLs (*p* = 0.0184) compared to *RNASEH2A*-mutated NT LCLs and was profoundly significant in *RNASEH2B*-mutated Met LCLs compared to *RNASEH2B*-mutated NT LCLs (*p* < 0.0001) (Figure 1B,C). These results strongly suggest that metformin effectively contributes to restoring mitochondrial health by ameliorating the altered membrane potential in AGS patient-derived LCLs.

Conversely, the JC-1 red signal, indicative of dye aggregation and a healthy mitochondrial membrane potential, displayed a distinct spot pattern. In both *RNASEH2B*-mutated NT and *RNASEH2A*-mutated NT LCLs, the aggregate red signal of JC-1 was significantly lower compared to healthy control NT LCLs (*p* = 0.0001 and *p* = 0.0003, respectively) (Figure 1B,D). Interestingly, after 72 h of metformin treatment, the mitochondrial membrane potential was significantly restored in both mutated LCLs (*p* < 0.0001 for both *RNASEH2B*-mutated Met and *RNASEH2A*-mutated Met LCLs when compared to their respective NT controls) (Figure 1B,D). To provide a more immediate and quantitative representation of the altered MMP in AGS patients and its subsequent restoration following metformin treatment, we calculated and graphed the ratio of red to green JC-1 fluorescence in Figure 1E.

Specifically, the ratio between mitochondria with a healthy potential (red fluorescence) and those with an altered potential (green fluorescence) was significantly lower in LCLs derived from both *RNASEH2B*-mutated NT and *RNASEH2A*-mutated NT patients compared to healthy controls. Following 72 h of metformin treatment, this ratio was significantly higher in the treated, mutated LCLs when compared to their untreated counterparts, unequivocally indicating the beneficial effect of the treatment on MMP.

These results collectively demonstrate that metformin treatment effectively restores the physiological mitochondrial membrane potential and exerts a beneficial effect on the observed mitochondrial alterations.

### 3.3. Metformin Treatment Reduces Stress-Induced Mitochondrial Damage via FOXO3 in RNASEH2B- and RNASEH2A-Mutated LCLs from AGS Patients

**Figure 2:**
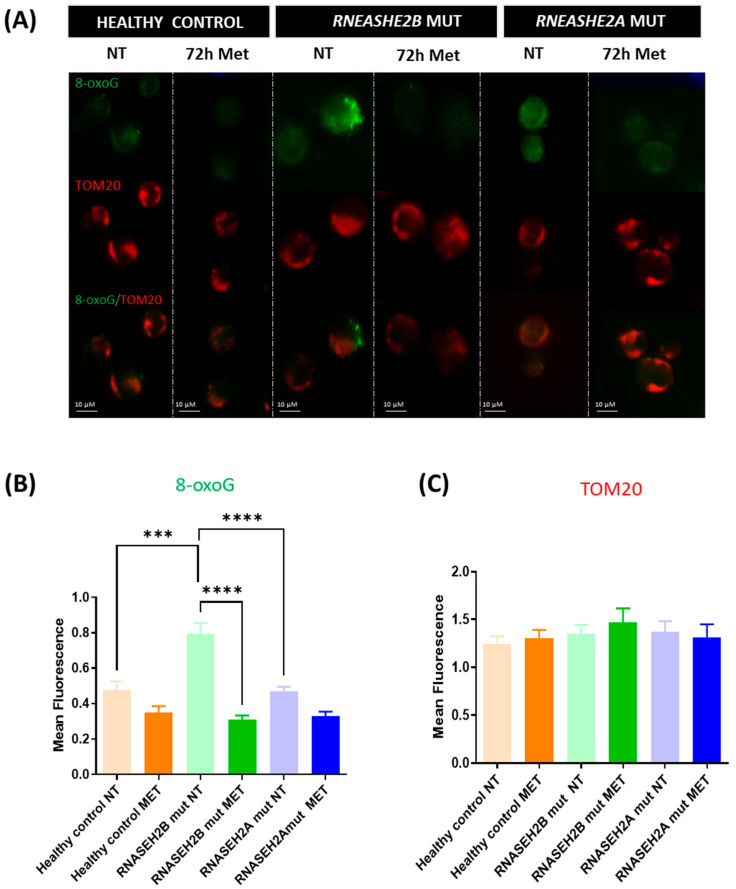
Oxidative stress-induced DNA damage in RNASEH2-mutated LCLs and healthy control LCLs. (**A**–**C**) Immunofluorescence and mean fluorescence of LCLs derived from a healthy control and AGS patients with mutations in the *RNASEH2B* and *RNASEH2A* genes with (72 h Met) or without (NT) 72 h metformin treatment, stained with an 8-oxoG antibody (green) and a TOM20 antibody (red). The bar graph represents the mean of three biological experiments. Three cells per image were quantified, and three images per condition were analyzed. Two-tailed Student’s *t*-test was performed, and data are expressed as means ± SEM; *** *p* < 0.001; **** *p* < 0.0001.

To analyze the impact of increased ROS production on oxidative stress-induced DNA damage, specifically represented by 8-oxoG levels, we performed IF analysis. This involved using a specific monoclonal anti-8oxoG antibody alongside an anti-TOM20 antibody for mitochondrial staining (Figure 2A–C).

The 8-oxoG signal was significantly higher in the *RNASEH2B*-mutated NT LCLs when compared to both *RNASEH2A*-mutated NT LCLs and healthy control NT LCLs (*p* < 0.0001 and *p* = 0.0003, respectively) (Figure 2A,B). Interestingly, 72 h of metformin treatment significantly reduced the 8-oxoG signal in *RNASEH2B*-mutated Met LCLs (*p* < 0.0001) (Figure 2B). Conversely, TOM20, which stains the mitochondrial compartment and was used as marker for the total amount of cellular organelles, did not show any significant differences among the cell lines (Figure 2C).

**Figure 3:**
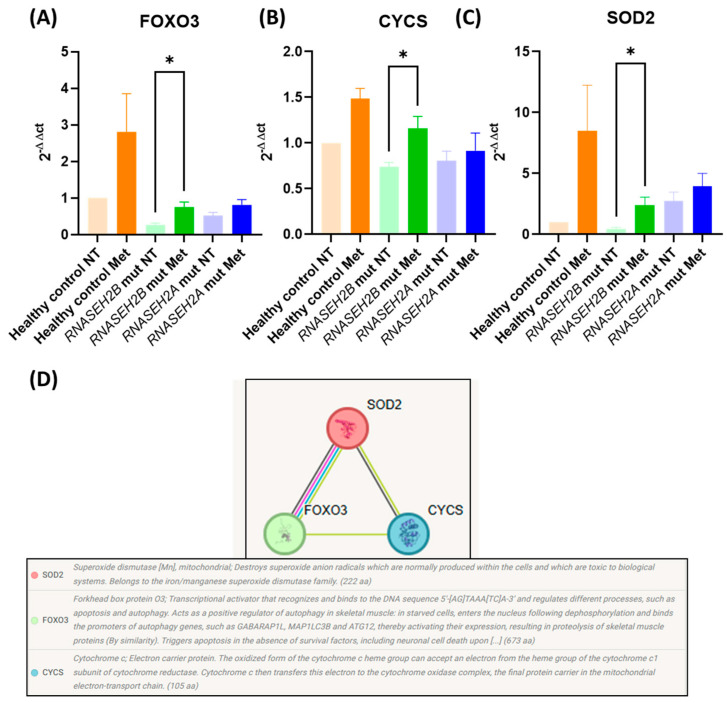
mRNA expression levels of *FOXO3*-dependent pathway components in RNASEH2 mutated LCLs and healthy control LCLs. (**A**–**C**) *FOXO3*, *CYCS*, and *SOD2* mRNA expression levels evaluated by RT-qPCR and normalized to the *β*-*actin* gene in *RNASEH2B*- and *RNASEH2A*-mutated LCLs in the presence (72 h Met) or absence (NT) of 72 h metformin treatment. The bar graph represents the mean of three biological experiments (n = 3). Two-tailed Student’s *t*-test was performed, and data are expressed as means ± SEM; * *p* < 0.05. (**D**) Representation of the *FOXO3* pathway and its interactions, constructed using STRING-pathway version 11.5.

Given that metformin has been shown to mitigate oxidative stress in immune cells by activating the *AMPK* and *FOXO3* pathways [19], we evaluated its effect on three key genes within this signaling cascade. We performed RT-qPCR to assess the mRNA expression levels of *FOXO3*, *CYCS*, and *SOD2*, all of which are involved in the FOXO3-dependent pathway (Figure 3D).

As shown in Figure 3A,B, both *FOXO3* and *CYCS* were downregulated in the NT LCLs from both RNASEH2-mutated patient cell lines compared to healthy controls, though these differences did not reach statistical significance.

However, after 72 h of metformin treatment, we observed increased expression of all analyzed genes. This led to statistically significant upregulation of *FOXO3* (*p* = 0.016), *CYCS* (*p* = 0.023), and *SOD2* (*p* = 0.022) mRNA levels specifically in the *RNASEH2B*-mutated Met LCLs.

These findings suggest that the beneficial effects of metformin in reducing ROS and restoring oxidative stress conditions may be directly linked to upregulation of the FOXO3-dependent signaling pathway, which notably includes well-known ROS scavenger genes like *SOD2*.

## 4. Discussion

Our previous work [17] demonstrated the presence of morphological, functional, and metabolic alterations in the mitochondria of immortalized lymphoblasts derived from AGS patients carrying *RNASEH2B* and *RNASEH2A* mutations. These findings led us to hypothesize a putative role for mitochondrial dysfunction in AGS pathogenesis, particularly highlighting increased oxidative stress in *RNASEH2B*-mutated LCLs.

In this context, metformin, a biguanide drug that has long been used to treat type 2 diabetes due to its robust glucose-lowering effects, well-established safety profile, and relatively low cost, is known to activate the *AMPK*- and *FOXO3*-dependent pathways [19,20,23,24]. Metformin treatment has been shown to restore mitochondrial membrane potential, promote mitochondrial network formation, and reverse mitochondrial ultrastructure abnormalities [20]. Furthermore, metformin treatment decreases interleukin (IL-1β) expression during prolonged exposure to pro-inflammatory stimuli by lowering ROS levels [22,23].

In the present work, we aimed to explore the beneficial effects of metformin treatment on *RNASEH2B*- and *RNASEH2A*-mutated LCLs, specifically focusing on its ability to reduce ROS production and reverse the oxidative stress in LCLs derived from AGS patients. Given the rarity of AGS and the inherent challenges in consistently obtaining fresh blood samples from pediatric patients for research purposes, we strategically chose to utilize immortalized cell lines. This decision not only ensures a continuous source of experimental material, but also provides design continuity with our initial published work on these same cell lines. Moreover, as AGS is characterized by a significant inflammatory component, the use of immortalized lymphoblasts offers a promising avenue for future studies, allowing for thorough evaluation of various interventions on the inflammatory response itself.

### 4.1. ROS Production

Our findings initially demonstrate that metformin treatment significantly reduces ROS generation in *RNASEH2B*-mutated LCLs, which, in their untreated state, exhibited significantly higher ROS levels compared to both healthy control LCLs and *RNASEH2A*-mutated LCLs (NT). This observation is consistent with the existing literature, where metformin treatment has been shown to powerfully diminish ROS release and favorably modulate oxidative stress-related environments [17,25,26]. These results align with the known phenotypic features of *RNASEH2B*-mutated AGS patients, who are typically characterized by a more pronounced and severe clinical condition compared to those with other mutations [6,7].

### 4.2. Mitochondrial Membrane Potential

Maintaining a negative MMP is critical for proper mitochondrial function [27]. Our analysis of JC-1 green fluorescence, which indicates organelles with altered MMP, revealed significantly higher levels in *RNASEH2B*-mutated NT LCLs, corroborating our previous work [17]. Crucially, metformin treatment led to a significant reduction in this green signal, indicating recovery of a physiological MMP. A similar, though less pronounced, trend was observed for *RNASEH2A*-mutated LCLs following metformin treatment. Conversely, JC-1 red fluorescence, indicative of a physiological mitochondrial membrane state, was significantly lower in both *RNASEH2B*- and *RNASEH2A*-mutated NT LCLs compared to healthy control NT LCLs. Upon metformin treatment, this red fluorescence was markedly increased in both AGS LCL types. Metformin’s capacity to restore the physiological membrane potential in dysfunctional mitochondria has been previously substantiated in various contexts [20,28], underscoring its potential utility in re-establishing fundamental mitochondrial health.

### 4.3. Stress-Induced DNA Damage

Metformin’s ability to reduce ROS levels and restore mitochondrial membrane potential underscores its beneficial effect in attenuating oxidative stress. Among the deleterious consequences of an oxidative stress-associated environment and ROS release, guanine is the most susceptible nucleobase to oxidation, resulting in the formation of 8-oxoG. This adduct can form at the DNA, RNA, or free nucleotide level and, if incorporated during RNA transcription or DNA replication, can lead to underlying epigenetic changes [29]. Our previous work [17] demonstrated elevated 8-oxoG levels in mutated LCLs, particularly in *RNASEH2B*-mutated LCLs, highlighting the impact of ROS overproduction on DNA integrity.

In the current study, we further confirm this finding in *RNASEH2B*-mutated NT LCLs, which exhibited significantly higher levels of 8-oxoG signal compared to *RNASEH2A*-mutated NT LCLs and healthy control NT LCLs. Although metformin treatment consistently yielded an improving trend in 8-oxoG levels, and consequently in ROS-dependent DNA damage, in both *RNASEH2B* and *RNASEH2A* mutants, it is crucial to highlight the marked difference in basal 8-oxoG levels between the two mutated AGS patient cell lines in the NT condition. This observation aligns with our previous findings [17] and reflects the fact that, despite carrying a compound mutation involving the catalytic subunit of this heterotrimeric complex, *RNASEH2A*-mutated AGS patients often exhibit a clinical phenotype more closely resembling that of healthy controls, though the underlying reasons are not yet fully understood.

As anticipated, oxidative stress-induced DNA damage was strongly reduced after metformin treatment, indicating the biguanide molecule’s capacity not only to act as a ROS scavenger, but also to modulate DNA damage. This is consistent with research suggesting that metformin can influence chromatin changes induced by metabolites and affect the metabolo-epigenetic apparatus [30]. Metformin’s multifaceted ability to link metabolic signals to chromatin state underlies much of its capacity to alter the epigenetic landscape [30].

### 4.4. Antioxidant Pathways

Considering metformin’s established ability to reduce ROS production and release, we subsequently assessed the expression of three antioxidant genes involved in the *FOXO3* pathway. In the NT condition, metformin treatment led to increased expression levels of all three genes (*FOXO3*, *CYCS*, and *SOD2*) in both *RNASEH2B*- and *RNASEH2A*-mutated LCLs compared to healthy control LCLs. Notably, only the treated *RNASEH2B*-mutated LCLs achieved statistical significance for these changes. The involvement of the *FOXO3*-dependent pathway in reducing oxidative stress is well-documented [19]. We hypothesize that this pathway can be activated by metformin in LCLs from AGS patients, thereby acting as an antioxidant mechanism that favors the re-establishment of a non-stressed environment, leading to normal mitochondrial function.

However, this remains a hypothesis, and broadening the patient cohort is necessary to confirm these results. Furthermore, additional studies will be valuable to more comprehensively understand the impact of metformin treatment on the metabolic and energetic alterations previously described for AGS-derived LCLs, as well as its effects on mtDNA release and the IFN-α mediated immune response.

## 5. Conclusions

Although the results of this work originate from a limited cohort of two patients, one carrying a mutation in the *RNASEH2B* gene and the other in the *RNASEH2A* gene, they appear promising for future studies and offer insights into the possible use of metformin in the treatment of AGS patients. Metformin’s impact in modulating oxidative stress, reducing ROS production, and decreasing stress-dependent DNA damage suggests that the drug is a good candidate for therapy for affected children.

Future research should focus on investigating the role of this drug in modulating the inflammatory response and the release of IFN-α, given that these are major features of the pathology. Furthermore, it will be crucial to evaluate metformin’s ability to restore the physiological morphology and functionality of altered mitochondria, particularly in *RNASEH2B*-mutated patients.

## 6. Limitations of the Study and Future Perspectives

We identified different limitations within the present work. Firstly, AGS is a rare disease, often affecting very young children, sometimes as early as at birth. Consequently, the study of its molecular etiology is inherently constrained by the challenges of patient recruitment. In this study, we utilized immortalized LCLs derived from two patients: one with an *RNASEH2B* gene mutation and one with an *RNASEH2A* gene mutation. This approach was necessitated by the impracticality of obtaining fresh blood samples from these pediatric patients with the periodicity required for our experimental plan. Continuous access to new samples or recruitment of additional cases is severely limited, particularly given the restricted AGS caseload in Italy [7].

Furthermore, it is important to note that this work serves as a continuation of our previous study [17] on mitochondrial dysfunction, which involved the same three cell lines used for the drug treatments investigated here. An additional limitation pertains to the choice of the healthy control. While their age does not precisely match that of the AGS patients, we selected the youngest available control for this investigation, as we are unable to collect samples from healthy subjects under 18 years of age. Moreover, AGS is a neuroimmune disorder characterized by a strong inflammatory component, precluding the use of patients with other diseases as study controls, as any confounding clinical aspect could influence and potentially induce an inflammatory response.

For future studies, our primary interest lies in expanding the patient cohort to further test the drug’s efficacy. This expanded evaluation will encompass not only the oxidative aspect, but also various inflammatory pathways that may benefit from metformin treatment. From this pilot study, it emerged, as expected, that metformin’s effect appears to be primarily centered on the *RNASEH2B*-mutated cell line. This result aligns with our previous investigation, which consistently showed that *RNASEH2B*-mutated patients exhibiting the worst pathological condition in terms of oxidative stress-induced DNA damage, morphological alterations, and ROS production [17]. Our future objective will be to delve deeper into the effects of this treatment on patients with mutations in the *RNASEH2B* gene, specifically evaluating any differences in efficacy based on the two distinct phenotypes (one milder, one more severe) that can be associated with the same p.A177T mutation in this gene.

## Figures and Tables

**Table 1:** Clinical and demographic data of the study cohort [7].

Sample	Gene	Gender	Variant	Amino Acid Substitution	Population Frequency	Disease Onset	Treatment at Sampling	Age at Sampling	Clinical Phenotype	Chilblains and/ or Recurrent Fevers	Epilepsy
Pt1	*RNASEH2B*	male	c.529G>A	p.A177T	59%	Infantile	None	9	Tetraparesis	Yes	Yes
Pt2	*RNASEH2A*	female	c.322C>T + c.690C>A	p.R108W + p.F231L	4%	Prenatal/neonatal	None	13	Spastic-dystonic tetraparesis	No	No
HC	Na	female	Na	Na	Na	Na	None	23	Na	Na	Na

Pt: patient; Na = not applicable; HC = healthy control.

**Table 2:** Primers sequences.

Gene	Primer Forward	Primer Reverse
*FOXO3*	GCAAGCACAGAGTTGGATGA	CAGGTCGTCCATGAGGTTTT
*CYCS*	TGGTTTTCTAGGACTGCCTCA	ACCTTGTGCTCACCATCTCC
*SOD2*	TTTTGAAATTCTATCTGTTGCTTGA	CCCCTGGAATTAAAACAGGA
*β*-*actin*	AGAGCTACGAGCTGCCTGAC	AGCACTGTGTTGGCGTACAG

## Data Availability

The datasets for this study can be found in the Zenodo repository (DOI: 10.5281/zenodo.8178044).
